# The impact of family urban integration on migrant worker mental health in China

**DOI:** 10.3389/fpubh.2024.1392153

**Published:** 2024-08-27

**Authors:** Xiaotong Sun, Mi Zhou, Li Huang

**Affiliations:** ^1^College of International Business, Shenyang Normal University, Shenyang, Liaoning, China; ^2^College of Economics and Management, Shenyang Agricultural University, Shenyang, Liaoning, China

**Keywords:** migrant workers, family urban integration, mental health, depressive symptoms, machine learning

## Abstract

**Background:**

As China has undergone the processes of urbanization and economic development, a large migrant population has emerged, creating new family migration trends. Family migration brings about changes in urban integration costs and benefits, affecting health investment.

**Objective:**

The primary objective of this research is to investigate the influence of urban integration of migrant workers' families on their mental wellbeing, with the aim of offering policy recommendations conducive to the realization of a comprehensive public health strategy in China.

**Methods:**

This paper uses multi-dimensional indexes to measure family urban integration, covering economic, social and psychological dimensions, which may consider the complexity of integration. Utilizing a machine learning clustering algorithm, the research endeavors to assess the level of urban integration experienced by migrant workers and their respective families. The analysis discerns three distinct clusters denoting varying degrees of urban integration within these familial units, namely high-level, medium-level, and low-level urban integration. We applied binary logit regression models to analyze the influence of family urban integration on the mental health among migrant workers. Then we conducted a series of robustness tests.

**Results:**

The results show that family urban integration decreases the probability of depressive symptoms by 14.6 percentage points. Further mechanism tests show that family economic integration enhances the psychological wellbeing of migrant workers by elevating their income status. Family social integration decreases depressive symptoms by increasing social status. Family psychological integration increases the psychological health of migrant workers by making them more satisfied with their lives. The heterogeneity test shows that family urban integration and its different dimensions have a strong impact on the depressive symptoms of women, first-generation, and less-educated groups.

**Conclusions:**

This study finds that family urban integration and its economic, social, and psychological dimensions significantly reduced the depressive symptoms of migrant workers. The results of this study lead the authors to recommend formulating a family-centered policy for migrant workers to reside in urban areas, optimizing the allocation of medical resources and public services, and improving family urban integration among migrant workers in order to avoid mental health problems in the process of urban integration.

## 1 Introduction

China has witnessed a substantial surge in urbanization since the 1980s, which has been accompanied by a substantial migration of individuals moving from rural regions to urban hubs. About 295.6 million migrant workers left their country hometown to look for better job opportunities in urban cities in 2022 (1). In this paper, the term “migrant workers” pertains to individuals who possess rural registration in China but relocate from rural areas to urban cities in different regions of China in search of employment (2, 3). The rise in population mobility has sparked academic interest in the mental health of migrants in host cities (4). Migrants from rural areas were discovered to be more prone to encountering prejudice and stigmatization in urban locales they inhabited (5). Rural-urban migrants also reported experiencing social and spatial exclusion (6). The level of urban integration they experience in host cities is frequently challenged, resulting in an increased vulnerability to depression and other mental health issues (7). At the same time, migrants are compelled to reside in specific areas of the city, mainly suburban regions and “urban villages”, where inadequate housing conditions prevail due to the hukou system's regulations (8). The absence of a native hukou exerts a negative effect on urban integration and contributes to a higher prevalence of mental health issues.

In sociology, the term “integration” initially referred to the cohesive force required by a society to address conflicts and disparities. While a consensus on the formal definition of integration remains elusive, the term is commonly employed to depict a reciprocal process of mutual adaptation, wherein both migrant individuals and resident populations are involved across various autonomous and interdependent domains. Heckmann defines integration as “a long-lasting process of inclusion and acceptance of migrants in the core institutions, relations and statuses of the receiving society” and “an interactive process between migrants and the receiving society, in which, however, the receiving society has much more power and prestige” (9). Heckmann proposed a conceptual framework for organizing integration processes, which includes structural integration, acculturation, interactive integration, and identification integration.

Within China, the notion of urban integration is perceived slightly differently. The majority of existing studies focus on a single dimension in order to draw implications for specific policy design. For instance, parameters such as income, education level, land utilization, and agricultural advancement were commonly employed as single-dimensional indicators (10–13). However, relying solely on one dimension for classification is insufficient in addressing the diverse and complex nature of urban integration. In light of this aspect, certain scholars attempted to achieve classification by constructing a comprehensive index that combines social, economic, public service, and environmental factors. For example, Wang et al. note that the integration of migrants in urban areas encompasses economic, social, cultural, and psychological aspects (14). Nevertheless, the accuracy of the weights allocated to each metric may be subject to a degree of subjectivity., significantly impacts the outcomes. Partitioning around medoids (PAM) is an effective technique for clustering data with multiple dimensions. It is capable of objectively identifying various family urban integration types, maximizing the heterogeneity between types and ensuring homogeneity within types. Additionally, it can address potential bias of researchers toward specific topics by incorporating multidimensional metrics. To address this research void, the current study intends to objectively classify various dimensions of urban integration typologies standpoint, utilizing an unsupervised machine learning approach.

Urban integration reflects the breadth and depth of migrants' engagement in urban societal activities. Urban integration can only be fully attained when migrants are psychologically and behaviorally integrated into mainstream society (15). Scholars widely agree that with the rise of urbanization, the split-household arrangement often evolves into family migration. Specifically, as urbanization intensifies, family reunification emerges as a prominent motive for migration (15). Family migration, regarded as the most established form of migration, has emerged as a prominent trend among migrant populations in China (16). Data shows that in 2010, in the host city, 70% of migrants resided with multiple family members, with over 28% of this group cohabiting with all family members (15). As per the findings delineated in the Report on China's Migrant Population Development, migrant families typically comprised an average of 2.61 individuals residing in their destination urban centers (17). On the whole, the family urban integration of migrant workers serves as the primary catalyst for fostering a people-centered approach to new urbanization (15).

Stark places the family, rather than the individual, at the center of the migration decision (18). Family sociology has extensively explored the varying degrees of family role dynamics in resource provision, a perspective corroborated by ethnographic studies on migration (19). Therefore, this study mainly focuses on how family urban integration affects the health of migrant workers.

## 2 Theoretical frameworks

The determinants of mental health are exceedingly intricate, influenced by various stages of social milieu and material economy (20). Research indicates that mental wellbeing correlates with physical health factors, including conditions like heart disease, hypertension, and diabetes, as well as socioeconomic indicators such as educational attainment, marital status, and personal and familial income levels (21–23). Particular attention be given to the influence of family migrant on health, since family migrant affects mental health not only immediately impact mental health, but also immediately through physical health condition and other socio-economic factors. The Grossman model of health capital (24, 25) furnishes a conceptual framework for examining the correlation between family migration and mental health outcomes.

Health economics research posits that the family constitutes a crucial determinant of health, including general economic circumstances; living and working conditions; as well as social and community networks (26), with a notably discernible correlation between family migration and health outcomes (27). Factors such a living in the host country for less than 2 years, insufficient family income, poor social support, and marital relationships were significantly associated with migrants' depression (28). While the debate persists regarding the directional impact of family migration on health within the theoretical framework of health economics, empirical investigations in this domain have substantiated the direct or indirect influence of family migration on health (29, 30). For instance, prior research has indicated that migrants residing with their families, particularly those cohabiting with their entire family, tend to exhibit superior socioeconomic status and maintain greater stability in their lives compared to migrants living alone (27). One study found that migrants are more likely to rely on family support for mental health issues (31). On the contrary, existing research indicates that migrants in different countries encounter significant stressors, including family poverty, housing instability, and employment challenges (32) that can affect their mental health. Therefore, existing research provides a robust theoretical foundation for analyzing the association between family migrant and unhealthy emotions.

Base on Grossman's framework, research indicates a correlation. Between family migrant and mental health. Specifically, the impact of family migration on mental health can manifest through three distinct pathways: (a) economic status; (b) social status; and (c) psychological support.

First, recent studies have underscored the significance of family migration as a prevalent phenomenon. Migration has advanced from an initial stage when it primarily involved single or married men, to one when it was primarily couples, to, most recently, whole families (16). The economic level stresses the importance of conflicting interests of family members. The framework facilitates an examination of the allocation of investments in health capital among family members (33). The costs and benefits of migrant workers' family in urban will affect the distribution of household assets, thereby affecting individual health investments. On the one hand, With the increase in the number of family members residing, costs also rise, creating more psychological stress (34). On the other hand, numerous rural laborers depart from their hometowns and migrate to urban areas in pursuit of employment opportunities and higher income prospects (35). This economic base can guarantee employment, wages, occupational prestige, and vocational training for migrant workers in the labor market (36), further relieving economic pressure and improving mental health.

Second, the social capital and networks of migrant workers contribute significantly to information dissemination, as well as provision of social and emotional support. Social interaction and mutual respect are fundamental requisites, as forging intimate and trustworthy relationships is essential for garnering support and acknowledgment from others, thereby enhancing mental wellbeing (37). The social dimension emphasizes the significance of social assistance and resources in easing family stress (38). Family urban integration allows for more friends and social connections. Migrant workers have the opportunity to establish trust and acquire assistance from social networks, which in turn aids them in managing challenges and adversities encountered in both personal and professional spheres, thereby enhancing their capacity to adapt to urban life (39). However, during the early stages of migration, rural migrants depart from their established social networks in rural areas and encounter a novel environment characterized by differences in climate, language, housing, and dietary norms from what they are accustomed to, bringing new social stressors.

Additionally, psychological factors significantly influence the correlation between family migration and health. Family urban integration improved identity recognition for migrant workers, which creates a sense of belonging for migrant workers, thus promoting mental health. Should this trend persist, an increasing number of migrants may be inclined to establish permanent residence in their host cities (15). At the same time, family migration can reduce the spatial exclusion of migrant workers in the urban, and thus reduce the risk of depression (40). However, as the influx of family members into urban areas increases, the demand for urban accommodations and public services, particularly essential resources such as healthcare and education, escalates. These burgeoning demands impose significant pressure on migrant workers (16), which can worsen their mental health. Considering the varied findings in existing literature, the objective of this study is to ascertain the directional impact of family urban integration on the mental wellbeing of migrant workers.

## 3 Methods

### 3.1 Data origin and sampling

The pool dataset used for this study comes from the China Family Panel Studies (CFPS) data for the years 2012, 2016, and 2018. The CFPS is a nationally representative, longitudinal social survey that was launched in 2010 and is conducted biennially by the Institute of Social Science Survey (ISSS) at Peking University, China. The survey design is based on the Panel Survey of Income Dynamics (PSID), the National Longitudinal Surveys of Youth (NLSY), and the Health and Retirement Study (HRS) in the United States. It focuses on a range of topics related to educational outcomes, economic activities, migration, health, and family dynamics. The survey collects data at three levels: the individual, family, and community levels.

The CFPS surveyed respondents in sampling units in 25 provinces (all provinces except Xinjiang, Tibet, Qinghai, Inner Mongolia, Ningxia, and Hainan), a sampling frame that represents 95% of the Chinese population. To generate a nationally- and provincially-representative sample, the CFPS adopted a “Probability-Proportional-to-Size” (PPS) sampling strategy with multi-stage stratification and carried out a three-stage sampling process. The first stage was the Primary Sampling Unit, in which county level units were randomly selected. In the second stage, village level units (villages in rural areas and neighborhoods in urban areas) were selected. In the third stage, households from the village level units were selected according to the systematic sampling protocol of the study. All members present at the time of surveying in each household were interviewed.

**Figure 1:**
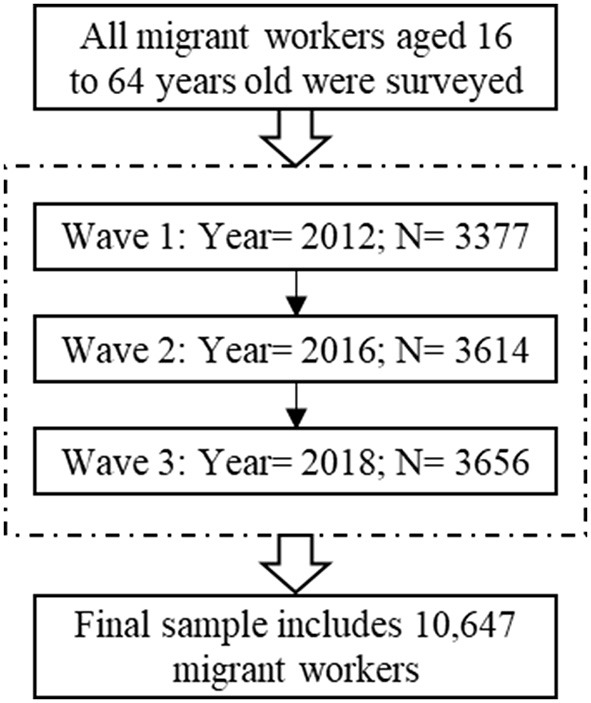
Flowchart of the sampling process.

Subsequent to an initial baseline survey conducted in 2010, Institute of Social Science Survey (ISSS) conducted five follow-up surveys in 2012, 2014, 2016, 2018, and 2020. For the aims of this study, we examine the family urban integration of migrant workers who were 16 to 64 years old at the time of the 2012, 2016, and 2018 surveys. For this study, we utilize CFPS data for the years 2012, 2016, and 2018, utilizing identical depression assessment tools, such as the Center for Epidemiologic Studies Depression Scale (CES-D), facilitates the amalgamation of datasets. This dataset comprises 10,647 migrant workers for 3 years, after removing observations with missing data (Figure 1, Supplementary Figure S1). Utilizing this pooled dataset enables us to investigate the depressive symptomatology experienced by migrant workers throughout these three time periods. The data underwent analysis utilizing Stata version 17.0.

### 3.2 Estimating depressive symptoms

Mental health refers to the condition of being able to face difficulties and pressures with optimism, and to work efficiently, reflecting mental pleasure and happiness (41). The mental health questionnaire employed in this study predominantly consists of items derived from the Center for Epidemiologic Studies Depression Scale (CES-D). The CES-D scale has been extensively utilized in the literature for assessing depressive symptoms (42). As shown in Supplementary Table S12, the CES-D scale comprises 20 items, with responses scored on a Likert scale, allowing respondents to indicate the frequency with which they experienced specific emotions over the past week.: “less than 1 day: score = 0”, “1–2 days: score = 1”, “3–4 days: score = 2”, and “5–7 days: score = 3”. There are two ways to look at mental health, positive and negative. Despite their interdependence, these variables can function independently (43). Sixteen items were used to assess the frequency of negative emotions, while four items were employed to investigate the occurrence frequency of positive emotions (questions 4, 8, 12, and 16 in Supplementary Table S12). Negative mental health refers to detrimental health issues, psychopathology, or psychiatric diseases. Positive mental health is defined as optimal mental functioning and a sense of wellbeing, such as eudaemonic and hedonic wellbeing (43). Thus, the scores of the four positive items were reversed, and subsequently summed to create a depression score variable. A higher score indicates a more pronounced presence of depressive emotions, reflecting poorer mental health status.

Scores on the scale range from 0 to 60. According to the US-based community mental health assessment survey, a score of 16 corresponds approximately to the 80th percentile of the CES-D distribution, so this threshold is often used to define depressive symptoms (44). Categorizing variables for depressive symptoms highlights qualitative distinctions, whereas regression analysis utilizing continuous variables may diminish the qualitative nuances. At the same time, considering that categorical variables may reduce regression power, we replace explained variable with continuous variables of CES-D scores in Supplementary Table S5 for robustness test. Consequently, we delineate the threshold at the 80th percentile, defining “depressive symptoms” as a CES-D total score of 16 or above. The Chinese adaptation of the CES-D scale has been extensively employed in prior research and has demonstrated reliability and validity within the Chinese demographic (45).

### 3.3 Estimating family urban integration

The majority of extant studies examining urban integration types tend to focus on a specific aspect (12, 46). However, restricting the classification of family urban integration types to a singular dimension is insufficient. Given that family urban integration is a multifaceted system with diverse objectives, any singular pursuit of developmental goals may disrupt the equilibrium and stability of the family urban integration index. The complexity and plurality inherent in the urban-rural system necessitate that family urban integration encompasses not only economic integration but also various aspects of demographic and societal integration (47, 48). Hence, a multidimensional perspective, incorporating comprehensive metrics delineating family urban integration, is imperative. Consequently, the primary objective of this study is to employ an unsupervised machine learning methodology to delineate family urban integration typologies based on multidimensional metrics encompassing economic, social, and psychological integration.

The paper identified family urban integration utilizing the partitioning around medoids (PAM) method from a multidimensional standpoint. The measurement software is Stata version 17.0, and the specific command is “cluster kmeans”. PAM serves as a robust tool for clustering multidimensional data. PAM, as a clustering algorithm, generates a cluster set as output with the objective of minimizing the average dissimilarity of objects to their respective cluster centers (49). This approach not only enables the objective identification of family urban integration types by maximizing heterogeneity between types and ensuring homogeneity within types, but also mitigates potential researcher bias toward specific topics by incorporating multidimensional metrics rather than relying on a single indicator. In practical terms, our study lays the groundwork for informing policy development related to family urban integration.

Based on previous studies (50, 51), our preliminary research, and the current situation in China, three dimensions were considered when examining family urban integration: economic, social, and psychological. Family economic integration represents a fundamental facet of family urban integration and serves as a precondition for achieving communal prosperity, typically characterized by income and expenditure patterns (52). Economic security has strong links to psychological stress in the literature (53). Economic disadvantage particularly affected some migrants; they believed it increased mental health need and served as a barrier to service access (54). Three variables are selected to estimate family economic integration: family income, family expenses, and the proportion of income spent on housing. Family social integration underscores equitable access to fundamental public services and the initiative to participate in social activities (48). Evidence suggests that the challenges associated with migration and resettlement can significantly impact mental health (55). Migrant can worsen mental health due to uncertain residency status, poor or limited access to services, and decreased opportunities to work or study (56–58). For example, tenure security, broadly defined as the right to government protection against forced evictions, can be compromised for those in the rental market who may be subjected to short-term leases or leases that do not protect against no cause evictions. This uncertainty and being forced to move can negatively impact social relationships and cause depression and anxiety (59, 60). Several studies have suggested that physical activities (61) and social interaction (62), potentially reduced the incidence of chronic diseases (63), increasing mental health. Nine variables are used to measure family social integration: property owned by family members, type of residential house, collectively-distributed land, popularity, membership in the Communist Party of China, membership in labor unions, membership in associations of individual workers, evaluation of the government, and donations. Family psychological integration reflects the self-identity approval of the migrants and their family members, and their willingness to migrate permanently to urban (64). Rural migrants experience homesickness and maintain cross-geographical ties with their family members and relatives, potentially leading to depression or illness (65, 66). By moving family members to the urban, along with the establishment of new social connections and emotional roots, rural migrants can develop place attachment in their new environment. This process can mitigate the adverse effects associated with mental stress (41). Six variables are selected to measure family psychological integration: probability and number of dependents moving with migrant workers, number of family members of having meals together, confidence in neighbors, and loneliness.

### 3.4 Statistical analysis

This paper used a machine learning clustering algorithm to assess the family urban integration of migrant workers. Specifically, it uses PAM clustering, with the Elbow method being used to determine the most suitable number of clusters. The PAM algorithm involves several steps: (1) Initial selection of k observations as medoids; (2) Assignment of each observation to the nearest medoid; (3) Iterative swapping of medoid and non-medoid observations to minimize dissimilarity cost; (4) Selection of the configuration with the lowest total dissimilarity; (5) Iterative repetition of steps two through four until medoids no longer change. Given the flexibility in determining the number of clusters, identifying the optimal number is crucial for accurate results (49). This study primarily employed the Elbow method, which assesses the within-cluster sum of squares, to ascertain the optimal number of clusters. The steepest decline in the percentage of explained variance by the clusters relative to the number of clusters occurred when the number of clusters equaled 3. We chose clusters = 3 to further divide family urban integration into three different tiers: low, medium and high (see Supplementary Table S2 for specific indicators).

The correlation between family urban integration and mental health can be identified in the following Equation 1:


(1) $$\begin{array}{l} D_{it}=\alpha +\beta_{1}I_{it}+\beta_{2}X_{it}+\beta_{3}H_{it}+\eta_{i}+\varpi_{t}+\varepsilon_{it} \end{array}$$

The explained variable *D*_*it*_ refers to mental health in the year *t* for person *i*. The main explanatory variables *I*_*it*_ are family urban integration and its economic, social and psychological dimensions. Variable *X*_*it*_ represents a range of individual attributes such as gender, age, and educational background, work for self/family, medical insurance, pension insurance and area fixed effect; *H*_*it*_ is a vector of household characteristics, including family size, education of spouse, education of father and education of mother; η_*i*_ is a fixed effect for person *i*; ϖ_*t*_ are survey wave indicators; and ε_*it*_ is an error term. Because mental health is a categorical variable, Logit was used to perform regression estimations on Equation 1.

Our study faces two primary challenges to result validity. Firstly, while migrant worker fixed effects address time-invariant unobserved heterogeneity, the presence of time-variant unobserved heterogeneity might persist, potentially biasing estimates. Put differently, factors unaccounted for in the model, such as personality traits, life experiences, and social networks, could influence both mental health and family urban integration, thus introducing potential bias into the results. Therefore, the calculations hinge on the presumption that the patterns in mental health outcomes for migrant workers whose family urban integration in each period remains constant (an assumption known as the parallel trend hypothesis). To examine the validity of the common trend assumption, this study compares the trends in each of the outcome variables among migrant workers with different levels of family urban integration in the periods before year *t* by re-estimating Equation 1, and incorporating an indicator for family urban integration in the subsequent wave following integration in the initial period *t*. Since the CFPS data is surveyed biennially, *l* has values of 2, 4, and 6 [see Equation 2]:


(2) $$\begin{array}{c} \begin{array}{c} D_{it}=\alpha +\beta_{0}I_{i\left(t+l\right)}+\beta_{1}I_{it}+\beta_{2}X_{it}+\beta_{3}H_{it}+\eta_{i}+\varpi_{t}+\varepsilon_{it},\\ l=2,\;4,\;6 \end{array} \end{array}$$

Second, a significant empirical obstacle in identifying the causal effect of family urban integration on mental health is that mental health may have a considerable influence on family urban integration.

To avoid reverse causation and attain unbiased and stable estimates, we assess community-level family urban integration and employ this variable as a proxy for actual family urban integration. Community-level family urban integration means family urban integration of other samples in the same village except oneself. “Community” refer to the place of residence of the migrant workers. The construction of community-level IVs includes only the migrant worker population. Specifically, we used the following four steps to generate instrumental variables. Firstly, calculate the sum of family urban integration of all sample in the same village by identifying village community ID (Define as total_integration). Secondly, compute the aggregate number of samples in the same village (Define as number_integration). Thirdly, calculate the total family urban integration of other samples in the same village excluding oneself (Define as other_integration). Fourthly, family urban integration of other samples in the same village except oneself is other_integration divided by (number_integration-1). IV of family economic, social and psychological integration used the same calculation method. Theoretically, these instrumental variables satisfy the conditions for being a credible IV for family urban integration. On the one hand, family urban integration of other samples in the same village except oneself typically signifies the social connections of migrants originating from the same region. Elevated levels of family urban integration frequently signify enhanced and broader social support networks, which in turn may lead to increased job opportunities, diminished migration expenses, and the facilitation of migrant employment (67, 68). On the other hand, it is presumed that family urban integration of other samples in the identical rural community except oneself does not have a direct impact on individual's own mental health (69).

We use the maximum likelihood estimator computational method to identify the IV estimates. The corresponding first stage equation of the IV estimations can be identified with the following Equation (3):


(3) $$\begin{array}{l} I_{it}=\alpha +\beta_{1}{mean\text{\_}I}_{it}+\beta_{2}X_{it}+\beta_{3}H_{it}+\eta_{i}+\varpi_{t}+\varepsilon_{it} \end{array}$$

*I*_*it*_ indicates family urban integration of migrant workers *i* at the time of the survey *t*. The *mean*_*I*_*it*_ is an instrumental variable referring to community-level family urban integration. Variable *X*_*it*_ is a range of individual attributes such as gender, age, and educational background, work for self/family, medical insurance, pension insurance and area fixed effect; *H*_*it*_ is a vector of household characteristics, including family size, education of spouse, education of father and education of mother; η_*i*_ is a fixed effect for person *i*; ϖ_*t*_ are survey wave indicators; and ε_*it*_ is an error term.

We choose the income status, social status, and life satisfaction to test the mechanism of family urban integration on mental health. The corresponding first stage equation can be identified in Equation (1). The corresponding second and third stage equation can be identified in Equations (4, 5):


(4) $$\begin{array}{l} M_{it}=\alpha +\beta_{1}I_{it}+\beta_{2}X_{it}+\beta_{3}H_{it}+\eta_{i}+\varpi_{t}+\varepsilon_{it} \end{array}$$

(5) $$\begin{array}{l} D_{it}=\alpha +\beta_{1}^{\prime }I_{it}+\tau M_{it}+\beta_{2}X_{it}+\beta_{3}H_{it}+\eta_{i}+\varpi_{t}+\varepsilon_{it} \end{array}$$

*M*_*it*_ is a mechanism variable referring to income status, social status, or life satisfaction. *I*_*it*_ indicates family urban integration of migrant workers *i* at the time of the survey *t*. *D*_*it*_ refers to mental health in year *t* by person *i*. Variable *X*_*it*_ is a range of individual attributes such as gender, age, and educational background, work for self/family, medical insurance, pension insurance and area fixed effect; *H*_*it*_ is a vector of household characteristics, including family size, education of spouse, education of father and education of mother; η_*i*_ is a fixed effect for person *i*; ϖ_*t*_ are survey wave indicators; and ε_*it*_ is an error term.

## 4 Results

### 4.1 Descriptive statistical analysis

The descriptive statistics for all samples can be found in Supplementary Table S1. The mean CES-D score for migrant workers was 12.01, with 29% exhibiting symptoms of depression. In terms of family urban integration, the average level for migrant workers was 1.305. To be specific, the family economic integration level of migrant workers was 1.277, the family social integration level was 1.759 and the family psychological integration level was 1.835. All of these values were below the medium level.

The regression analysis incorporates control variables encompassing the individual, familial, and social attributes of migrant workers., such as gender (Gender), age in years (Age), educational attainment (Illiterate/Semi-literate, Primary school, Junior high school, Senior high school, and 3-Year college or above), work status (Work for self/family, or for other individual/family/organization/company), use of medical insurance (Medical insurance), use of pension insurance (Pension insurance), educational attainment of spouse (Illiterate/Semi-literate, Primary school, Junior high school, Senior high school, and 3-Year college or above), educational attainment of parents (Primary school and below, Junior high school and above) and household size (Family size).

As is shown in Supplementary Table S1, 52.7% of the sample is male. In terms of education attainment, 17.7% are illiterate or semi-literate, 23% have a primary school education and 59.3% possess at least a junior high school level of education. The sample's mean age was 43.7 years. The proportion of migrant workers that work for self/family was 56.5%. The average number of medical insurance enrollees was 0.956, and the number of pension insurance enrollees was 0.651. Spouses of migrant workers had educational attainment similar to the migrant workers themselves. However, the educational level of their varies greatly, and their mothers were generally relatively uneducated. The average family size of the sample was 4.489 people.

### 4.2 Regression results

Supplementary Table S3 presents the principal findings regarding the influence of family urban integration on mental health outcomes. For each outcome, the coefficient and standard error on the family urban integration variable and its three dimensions are reported, as estimated utilizing two distinct variants of the Equation (1). We show the estimated effect of family urban integration using Logit without control variables (Column 1 and Column 2), and further controlling for additional migrant, household covariates, and regional fixed effects (Column 3 and Column 4). The first and second column of Logit correlation results offers indication of an inverse relationship between family urban integration and migrant depressive symptoms. The projected impacts of family urban integration on the depressive symptoms demonstrate a statistically significant negative association (at the 1% level) while adjusting for additional individual and household characteristics and regional fixed effects. Nonetheless, these estimates could be biased as they might not adequately consider significant sources of endogeneity.

Table 1 displays the primary estimates employing migrant fixed effects. After controlling for time-invariant heterogeneity, the influence of family urban integration on mental health demonstrates statistical significance. The results show that family urban integration decreases the probability of depressive symptoms by 14.6 percentage points. This outcome aligns with the discovery of Zhou et al. that the transition from rural to urban areas negatively impacts the mental wellbeing of migrants (71).

**Table 1:** Effect of family urban integration on depressive symptoms of migrant workers (Pooled Logit).

**Variables (definition)**	**Depressive symptoms (1** = **Yes; 0** = **No)**				
	**Two-way fixed effects**				
	**(1)**	**(2)**	**(3)**	**(4)**	**(5)**
Family economic integration^a^	−0.184^***^				−0.163^***^
(1 = low; 2 = medium; 3 = high)^b^	(0.050)				(0.050)
Family social integration		−0.307^***^			−0.243^***^
(1 = low; 2 = medium; 3 = high)		(0.034)			(0.035)
Family psychological integration			−0.309^***^		−0.237^***^
(1 = low; 2 = medium; 3 = high)			(0.035)		(0.036)
Family urban integration				−0.146^***^	
(1 = low; 2 = medium; 3 = high)				(0.046)	
Control variables^c^	Yes	Yes	Yes	Yes	Yes
Regional fixed effect	Yes	Yes	Yes	Yes	Yes
Year fixed effect	Yes	Yes	Yes	Yes	Yes
Migrant fixed effect	Yes	Yes	Yes	Yes	Yes
Observations^d^	10,647	10,647	10,647	10,647	10,647

Source: China Family Panel Studies (2012, 2016, and 2018).

^a***^Statistically significant at the 1% level; ^b^standard errors robust to heteroskedasticity are presented in parentheses; ^c^control variables include gender, age, age square, education, work for self/family, medical insurance, pension insurance, family size, and education of spouse, father and mother; ^d^we conduct regressions with weighted observations, prioritizing those less likely to remain in the sample, thereby aiming to preserve the sample's representativeness (70). Specifically, we calculate the wave number of participation in the survey, and then weight the regressions using the inverse of the calculated number.

Moreover, integration can weaken the impact.

The result is reasonable because the improvement of family urban integration can help migrant workers increase their economic and social status, and reduce loneliness, thus improving mental health. As documented in the literature, enhanced social integration typically contributes to the betterment of migrants' mental wellbeing. Specifically, factors such as economic integration, assimilation into the host culture, and psychological integration were found to correlate negatively with both psychological distress and perceived stress scores (71). For instance, Zhou et al. observed a reduction of 20.8 percentage points in the prevalence of depressive symptoms attributed to urban integration (72). This pattern is further corroborated by the data presented in Table 1.

A primary concern in estimating the effects of family urban integration on mental health outcomes is the endogeneity of integration. Stated differently, there could exist unobserved factors that are associated with both family urban integration and subsequently the mental health outcomes observed. Reverse causality might also be a plausible scenario.: if a migrant perceives themselves as being in poor health, he may be less likely to migrate or integrate into urban areas. Supplementary Table S4 shows that family urban integration is correlated with migrant depressive symptoms after controlling for other observable factors.

Table 2 shows the results of tests for parallel pre-trends. For each outcome, the pre-level of depressive symptoms is similar for different degrees of family urban integration in the current period, as denoted by the coefficients lacking statistical significance in family urban integration, *Family urban integration*_*i*(*t*+*l*)_, *l* = 2, 4, 6. The results offer partial validation for the assertion that reverse causality and the omission of time-related variables are improbable sources of bias affecting the primary outcomes.

**Table 2:** Tests for parallel pre-trends.

**Variables**	**Definition**	**Depressive symptoms (1** = **Yes; 0** = **No)**		
		**(1)**	**(2)**	**(3)**
Family urban integration _i(t+2)_^a^	1 = Low; 2 = Medium; 3 = High	−0.120		
		(0.092)		
Family urban integration _i(t+4)_	1 = Low; 2 = Medium; 3 = High		−0.052	
			(0.108)	
Family urban integration _i(t+6)_	1 = Low; 2 = Medium; 3 = High			−0.136
				(0.115)
Constant		1.159	−0.453	−1.771
		(1.028)	(1.134)	(1.278)
Control variables^b^		Yes	Yes	Yes
Regional fixed effect		Yes	Yes	Yes
Migrant fixed effect		Yes	Yes	Yes
Observations		2,178	1,750	1,530

Source: China Family Panel Studies (2012, 2016, and 2018).

^a^Standard errors robust to heteroskedasticity are presented in parentheses; ^b^control variables include gender, age, age square, education, work for self/family, medical insurance, pension insurance, family size, and education of spouse, father and mother.

Table 3 presents the estimated outcomes derived from the instrumental variable (IV) regression. Initially, in the first-stage estimation, we investigate the correlation between community-level family urban integration and the individual degree of integration (Table 3 Column 1-Column 4). Results for migrant workers indicate that community-level family urban integration increases personal integration by 76.3 percentage points. As indicated in the regression tables, the initial stage F-statistics for the instrumental variable exceed the typical threshold value for robust IV (i.e., 10), and they range from 27.09 to 180.4. Therefore, community-level family urban integration is strongly positively correlated with individual integration status. These results indicate that family urban integration at the county level is an effective instrument for enhancing the individual integration of migrant workers.

**Table 3:** Effect of family urban integration on depressive symptoms (instrumental variable regression).

**Variable definition (1 = Low; 2 = Medium; 3 = High)**	**First stage (1)–(4)**				**Second stage (5)–(8)**			
	**Family economic integration**	**Family social integration**	**Family psychological integration**	**Family urban integration**	**Depressive symptoms**			
	**(1** = **Low; 2** = **Medium; 3** = **High)**				**(1** = **Yes; 0** = **No)**			
	**(1)**	**(2)**	**(3)**	**(4)**	**(5)**	**(6)**	**(7)**	**(8)**
Community-level^a^ of integration^b^	0.799^***^							
	(0.022)							
Community-level of integration		0.450^***^						
		(0.037)						
Community-level of integration			0.237^***^					
			(0.032)					
Community-level of integration				0.763^***^				
				(0.024)				
Family economic integration					−0.293^***^			
					(0.079)			
Family social integration						−0.475^***^		
						(0.139)		
Family psychological integration							−0.529^**^	
							(0.236)	
Family urban integration								−0.212^**^
								(0.084)
F-statistics	180.40	27.09	158.67	133.25				
*p*-value	0.000	0.000	0.000	0.000				
Control variables^c^					Yes	Yes	Yes	Yes
Fixed effects^d^					Yes	Yes	Yes	Yes
Observations	10,647	10,647	10,647	10,647	10,647	10,647	10,647	10,647

Source: China Family Panel Studies (2012, 2016, and 2018).

^a***^/^**^Statistically significant at the 1%/5% level; ^b^standard errors robust to heteroskedasticity are presented in parentheses.; ^c^control variables include gender, age, age square, education, work for self/family, medical insurance, pension insurance, family size, and education of spouse, father and mother; ^d^fixed effects include regional fixed effect, year fixed effect and migrant fixed effect.

In the second stage, the impacts of family urban integration on mental health suggest that integration markedly alleviated the severity of depression (Table 3 Column 5-Column 8). The depressive symptoms of migrants with high levels of family urban integration are 21.2% points lower on average than that of low-integration individuals in the IV regression. The notable contrast between the IV estimates and the Logit estimates (14.6%) underscores the significance of employing the IV strategy to address endogeneity.

### 4.3 Robustness test

Due to the distribution characteristics of the explained variable, a dummy variable for depressive symptoms was replaced by CES-D scores for regression. Supplementary Table S5 illustrates the association between family urban integration and CES-D scores. Two-stage least-square regression results show that family urban integration significantly reduces the CES-D scores of migrant workers, this implies that the impacts on mental health remain consistent regardless of the type of dependent variable.

As shown in Supplementary Table S6, a robustness check is implemented to verify the persistence of the primary regression results. We employ a quantile regression model, with the CES-D score as the dependent variable. Supplementary Table S6 demonstrates that family urban integration consistently decreased the CES-D scores among migrant workers. The result is significant for any quantile. The research on the influence of family urban integration on mental health demonstrates robustness.

Machine learning demonstrates its efficacy in regression-based causal inference through the utilization of lasso to select instrumental variables (IV) for estimating the impact of family urban integration on mental health. The primary objective of machine learning is to enhance the precision of predicted values ($\hat{D}$). Yet as Belloni et al. observe, in any empirical analysis involving numerous covariates, it is essential to mitigate the risk of overfitting and the arbitrary findings associated with data mining (73). Similar concerns arise in causal models employing numerous instruments. Machine learning provides a useful guide to instrument selection, narrowing down a vast array of potential instruments, retaining only those demonstrating a robust initial stage. Supplementary Table S7 shows the regression results of IVlasso. The instrumental variables utilized are reflective of the community-level of family urban integration and its sub-indicators (see Supplementary Table S2 for detailed indicator characteristics). The results indicate that family urban integration and its three dimensions exert a notable adverse influence on the depressive symptoms experienced by migrant workers.

### 4.4 Intermediate outcomes

Table 4 presents fixed-effect estimates regarding the influence of family urban integration on intermediate outcomes. We find that improvement of family urban integration leads to an increase in income status, social status, and life satisfaction. To be more specific, we find a 15.5% increase in income status among migrant workers. We find a 23.2% gain in social status among migrants. We also find a 13.7% improvement in life satisfaction. These estimates imply that increases in income status, social status, and life satisfaction play a role in the positive effects of family urban integration on mental health found in Table 1.

**Table 4:** Fixed-effect estimates for the effect of family urban integration on intermediate outcomes.

**Variable (definition)**	**Income status**	**Social status**	**Life satisfaction**
	**(1** = **low-5** = **high)**	**(1** = **low-5** = **high)**	**(1** = **unsatisfied-5** = **satisfied)**
	**(1)**	**(2)**	**(3)**
Family economic integration^a^	0.155^***^		
(1 = low; 2 = medium; 3 = high)^b^	(0.023)		
Family social integration		0.232^***^	
(1 = low; 2 = medium; 3 = high)		(0.017)	
Family psychological integration			0.137^***^
(1 = low; 2 = medium; 3 = high)			(0.016)
Control variables^c^	Yes	Yes	Yes
Regional fixed effect	Yes	Yes	Yes
Year fixed effect	Yes	Yes	Yes
Migrant fixed effect	Yes	Yes	Yes
Observations^d^	10,647	10,647	10,647

Source: China Family Panel Studies (2012, 2016, and 2018).

^a***^Statistically significant at the 1% level; ^b^standard errors robust to heteroskedasticity are presented in parentheses; ^c^control variables include gender, age, age square, education, work for self/family, medical insurance, pension insurance, family size, and education of spouse, father and mother; ^d^we conduct regressions with weighted observations, prioritizing those less likely to remain in the sample, thereby aiming to preserve the sample's representativeness (70). Specifically, we calculate the wave number of participation in the survey, and then weight the regressions using the inverse of the calculated number.

In order to further elucidate the correlation between the intermediate outcomes and mental health of sample migrant workers, we conduct regressions of depressive symptoms on each intermediate outcome, controlling for all variables listed in Supplementary Table S1, alongside survey wave indicators, and both regional and individual fixed effects. The findings indicate statistically significant associations between intermediate outcomes and mental health outcomes (see Table 5).

**Table 5:** Intermediate outcomes and depressive symptoms (Pooled IVprobit).

**Variable (definition)**	**Depressive symptoms (1** = **Yes; 0** = **No)**		
	**(1)**	**(2)**	**(3)**
Family economic integration^a^	−0.281^***^		
(1 = low; 2 = medium; 3 = high)^b^	(0.082)		
Income status	−0.127^***^		
(1 = low-5 = high)	(0.014)		
Family social integration		−0.414^**^	
(1 = low; 2 = medium; 3 = high)		(0.169)	
Social status		−0.099^***^	
(1 = low-5 = high)		(0.024)	
Family psychological integration			−0.478^*^
(1 = low; 2 = medium; 3 = high)			(0.275)
Life satisfaction			−0.299^***^
(1 = unsatisfied-5 = satisfied)			(0.022)
Control variables^c^	Yes	Yes	Yes
Regional fixed effect	Yes	Yes	Yes
Year fixed effect	Yes	Yes	Yes
Migrant fixed effect	Yes	Yes	Yes
Observations	10,647	10,647	10,647

Source: China Family Panel Studies (2012, 2016, and 2018).

^a***^/^**^/^*^Statistically significant at the 1%/5%/10% level; ^b^standard errors robust to heteroskedasticity are presented in parentheses.; ^c^control variables include gender, age, age square, education, work for self/family, medical insurance, pension insurance, family size, and education of spouse, father and mother.

Subsequently, to test the robustness of the mechanism, we employed KHB mediation analysis to explore the effects of mediators in non-linear models. Given the non-linear (binary) characteristics of the dependent variables, the conventional mediation procedure (74) is unsuitable. Therefore, the KHB method proves to be particularly valuable in shedding light on the influence of mediators in such models. The KHB model quantifies total, direct, and indirect effects using the logit method. The total effects can be dissected into direct and indirect effects through comparison of the coefficients in the linear model. As shown in Supplementary Table S8, family urban integration had significant effects on depressive symptoms through income status, social status, and life satisfaction. For these factors, indirect effects accounted for 16%, 14% and 21%, respectively.

### 4.5 Heterogeneity analysis

To discuss the influence of family urban integration on mental health in different groups of migrant workers, we categorized the sample into groups according to gender, age, and education to examine the diverse impacts. First, the heterogeneity analysis based on gender is presented in Supplementary Table S9. Previous research (75) suggests that various factors, including biological (or hormonal) factors and social influences such as sexual discrimination, might contribute to women's increased susceptibility to depression. Therefore, family urban integration exerts a notable influence on mental health for women. For instance, Supplementary Table S9 indicates that family urban integration has a significant effect on depressive symptoms among women migrants, which means that integration may alleviate the effects of sexual discrimination on women. Supplementary Figure S2 also shows the same trend.

Second, the influence of family urban integration on mental health may vary according to age. Hsieh and Qin (44) have highlighted that older adults face a heightened risk of severe depression compared to younger individuals. This finding aligns with prior research indicating elevated rates of depression among older demographics (76). Consequently, we investigated the variation in the impact of integration on mental health across different age groups. As illustrated in Supplementary Table S10, Supplementary Figure S3, the adverse influence of family urban integration on psychological wellbeing was notable among first-generation migrants. One conceivable explanation is that first-generation migrants tend to experience lower social standing owing to their compromised health and limited social networks.

Finally, almost 40% of migrants in the CFPS sample have not completed junior high school. Educational attainment is highly correlated with a tendency toward depression (44). To examine potential variations in the observed impacts based on educational background, our analysis involved segmenting the migrant worker sample into three distinct groups., utilizing those who have at most a primary school education, those who only completed junior high school, and those who graduated from high school. Supplementary Table S11 indicates that family urban integration is most significant in alleviating depressive symptoms for those migrant workers who had only a primary school education. Li et al. found that the lower the level of education of migrant workers, the lower the level of urban integration (15). Education plays a pivotal role in facilitating the accumulation of human capital and fostering personal agency over life circumstances, potentially elucidating the heightened susceptibility to depression among individuals with lower levels of education. Therefore, family urban integration plays an important role in improving mental health in less educated groups. The same trend can be seen in Supplementary Figure S4.

## 5 Discussion

Utilizing nationally-representative CFPS pool datasets spanning from 2012 to 2018, this study demonstrates a noteworthy reduction in the occurrence of mental health issues among migrant workers in China attributed to family urban integration. These depressive symptoms can negatively impact work outcomes, as research has shown that individuals experiencing depression are more prone to subpar work performance (77). If depression rates are high nationwide in China, it could potentially affect the country's human capital. Moreover, depressive symptoms in adults have been strongly linked to criminal behavior and suicidal tendencies (78). Therefore, improving mental health can improve productivity, increase earnings, reduce criminal activity, and enhance public health. Specifically, the depressive symptoms of those workers with higher family urban integration are, on average, 14.6% lower than that of migrant workers with lower integration in the baseline estimations, and 21.2% lower in the IV regressions. The findings underscore the necessity of implementing measures to enhance the urbanization process. Thus, enhancing the mental health of migrant workers.

While family urban integration may positively impact the mental health of Chinese migrant workers, the results indicate that this influence differs significantly among specific subgroups. This divergence may be attributed to the distinct roles that family-related factors play within different demographic segments. For instance, the effect of family urban integration appears more pronounced on the mental wellbeing of women compared to men. In Chinese societal norms, women typically prioritize familial values over individualistic pursuits. In addition, women are more likely to be depressive than men (44). Therefore, the impact of family urban integration on psychological health is more pronounced in women. Additionally, first-generation migrant workers typically demonstrate stronger familial bonds (79). In terms of educational attainment, less educated populations have much higher incidences of depression, and family migration networks facilitate integration (80). It is therefore reasonable to believe that less-educated migrant workers may suffer from less depression when they are integrated into urban communities.

While existing studies have not conducted comprehensive examinations of the impact of family urban integration on depressive symptoms among subgroups of migrant workers in China, the findings presented in this study regarding the impact of integration on mental health are substantiated by existing literature. As an illustration, Raitakari et al. posit that migrants who experienced social isolation and infrequent engagement in social organizations or activities encountered adverse effects on their mental wellbeing (81). Moreover, migrants with a strong will to integrate could bring their family members to urban areas, thus, prolonged residence in urban areas may lead to enhancements in their health over time. In general, the integration process indeed contributed to the enhancement of various health dimensions among internal migrants.

This study possesses several strengths. Firstly, our sampling frame encompasses 95% of the Chinese populace, thereby warranting its national representativeness. Additionally, The CFPS, as an annual longitudinal survey, provides a valuable resource for constructing pooled data for analysis. Additionally, its substantial sample size of 10,647 participants enhances the statistical power and external validity of our research. Furthermore, all data were uniformly collected by the Institute of Social Science Survey (ISSS) at Peking University, ensuring consistency and reliability in the sampling approach. Lastly, this study centers on investigating the impact of family urban integration on depressive symptoms among various sub-groups. Analyzing various categories of migrants allows for a deeper understanding of the impact of family urban integration on mental wellbeing.

While our study possesses notable strengths, it is important to acknowledge its limitations. First, one such limitation is that the data collected by the CFPS relied solely on self-reported information, precluding the possibility of conducting medical evaluations to ascertain the depressive status of migrant workers. Second, while the CFPS data provides national representativeness and covers 95% of the population, there are certain limitations in the questionnaire items related to family urban integration. Future research could benefit from conducting in-depth social surveys specifically focused on studying family urban integration, in order to comprehensively consider variables that measure family urban integration. Third, we only conducted a current study and did not consider the impact of rural migrants' historical life experiences before entering the host city on their mental health. Further studies should construct a life-course framework to analyze the family migration effects on existing experiences or add a retrospective social survey. Subsequent investigations aiming to elucidate the causal relationship between family urban integration and depressive symptoms in China should address these aspects in greater detail.

That said, there are at least three future research directions. First, we used the CES-D scale to measure the mental health of migrant workers. However, the issues covered by this scale rely solely on self-reporting by respondents and do not involve medical diagnosis of depression. With the continuous development in the medical field, future research could utilize objective indicators to measure the prevalence of depression, thus enhancing the reliability of research findings. By combining medical diagnostic methods with psychological measurement tools, a more comprehensive assessment of respondents' depression status can be achieved, providing more accurate and scientific data support for in-depth exploration of the mental health issues of migrant workers. Future studies can focus on this direction to offer more targeted recommendations and interventions for policy-making and mental health interventions. Second, future research endeavors could focus on addressing the limitations identified in the current study regarding the measurement of family urban integration. Specifically, we could consider developing more comprehensive and tailored survey instruments that target the various dimensions of family urban integration. By incorporating a wider range of variables and utilizing qualitative research methods, such as in-depth interviews or focus group discussions, we can gain a deeper understanding of the complexities and nuances of family urban integration. Additionally, exploring the interplay between individual characteristics, family dynamics, and urban environments could provide valuable insights into how these factors measure the process of family urban integration. By adopting a more holistic and multi-faceted approach to studying family urban integration, future research has the potential to offer a more comprehensive understanding of this important phenomenon. Third, another important aspect for future research is to address the limitation of solely conducting a current impact. It is crucial to consider the influence of rural migrants' historical life experiences prior to migrating to the host city on their mental health. Subsequent studies could benefit from constructing a life-course framework to examine the impact of past family migration experiences on mental health or incorporating a retrospective social survey. By incorporating these elements into future research, a more comprehensive understanding of the long-term effects of family migration on mental health outcomes among rural migrants can be achieved, providing valuable insights for policy-making and intervention strategies in this area.

From a policy perspective, firstly, the government should give high priority to addressing the mental health issues of migrant workers, a vulnerable group in society that has been overlooked. Enhancing the spiritual wellbeing of urban communities necessitates a focus on the mental health of migrant workers. Specifically, government policies should incentivize and guide medical institutions to establish specialized mental health services for migrant workers. Moreover, community workers should provide regular psychological counseling to address the mental health needs of migrant workers. In addition, public awareness campaigns should encourage the families of migrant workers to prioritize their mental health and take measures to alleviate psychological stress. Future policies should emphasize improving the professionalism and accessibility of mental health services for migrant workers, while also enhancing societal awareness and support for their mental wellbeing. Secondly, the results of our study suggest that the Chinese government should prioritize the overhaul of its mental health and insurance mechanisms to eliminate the barriers imposed by existing policies. This aspect holds particular significance for migrant workers, who face significant challenges in accessing mental health resources. Specifically, to prevent an imbalance between the demand and the supply of medical services, it is necessary to increase the training of psychiatrists who can effectively address mental illness. Moreover, there exists a notable discrepancy in the standard of medical care among different regions across China. Hence, it is imperative for the government to establish a payment system that is suitable for local circumstances, in order to alleviate financial obstacles. By addressing depression promptly, it is possible to minimize the financial and social repercussions associated with it. Lastly, it is also important to have a family-centered perspective on improving social policies in the context of massive migration in China, including by adopting family-friendly migration policies and implementing social policies for improving family welfare and development for migrants. In addition, this study proposes that special consideration should be given to specific subgroups within the larger population, including women, first-generation migrants, and individuals with limited education. By doing so, it is believed that the family urban integration and the mental health of society can be improved.

## 6 Conclusion

Utilizing nationally representative datasets, this investigation delved into the correlation between family urban integration and the mental wellbeing of migrant laborers. Furthermore, it scrutinized the underlying mechanisms linking integration with depression, considering economic, social, and psychological factors. The findings revealed a consistently significant adverse association between family urban integration and the mental health of migrant workers. In particular, family urban integration and its different dimensions have a strong impact on the depressive symptoms of women, first-generation, and less-educated groups.

## Data availability statement

Publicly available datasets were analyzed in this study. This data can be found here: the data of the studies are publicly available and can be accessed via the website: China Family Panel Studies (CFPS). The datasets analyzed during the current study are available in the [CFPS] repository, http://isss.pku.edu.cn/cfps/index.htm. Readers can register on the CFPS website to access and download the data.

## Ethics statement

The studies involving humans were approved by “Peking University Biomedical Ethics Committee” Ethics Review Number: IRB00001052-14010, approved the study protocol. The studies were conducted in accordance with the local legislation and institutional requirements. Written informed consent for participation in this study was provided by the participants' legal guardians/next of kin.

## Author contributions

XS: Data curation, Formal analysis, Writing – original draft. MZ: Conceptualization, Methodology, Writing – review & editing. LH: Conceptualization, Writing – review & editing.
