# Supplementary material for: The impact of family urban integration on migrant worker mental health in China
Source: Front Public Health. 2024 Aug 27;12:1392153. doi: 10.3389/fpubh.2024.1392153 (PMC11385871; doi:10.3389/fpubh.2024.1392153)
Supplement: Supplementary file 1 [file Data_Sheet_1.docx]

Table S1 Sample summary statistics for key variables of the pool dataset

| Variables | Definition | Mean | S.D. | Min | Max |
| --- | --- | --- | --- | --- | --- |
| **Explained variables** |  |  |  |  |  |
| CES-D | Scores | 12.01 | 7.268 | 0 | 52 |
| Depressive symptoms ^a^ | 1=Yes; 0=No | 0.290 | 0.454 | 0 | 1 |
| **Explanatory variables** |  |  |  |  |  |
| Family urban integration | 1=Low; 2=Medium; 3=High | 1.305 | 0.548 | 1 | 3 |
| Family economic integration | 1=Low; 2=Medium; 3=High | 1.277 | 0.519 | 1 | 3 |
| Family social integration | 1=Low; 2=Medium; 3=High | 1.759 | 0.742 | 1 | 3 |
| Family psychological integration | 1=Low; 2=Medium; 3=High | 1.835 | 0.772 | 1 | 3 |
| **Instrumental variables ^b^** |  |  |  |  |  |
| Community-level of family urban integration | 1=Low; 2=Medium; 3=High | 1.305 | 0.209 | 1 | 3 |
| Community-level of family economic integration | 1=Low; 2=Medium; 3=High | 1.277 | 0.213 | 1 | 3 |
| Community-level of family social integration | 1=Low; 2=Medium; 3=High | 1.759 | 0.197 | 1 | 3 |
| Community-level of family psychological integration | 1=Low; 2=Medium; 3=High | 1.835 | 0.225 | 1 | 3 |
| **Mechanism variables** |  |  |  |  |  |
| Income level | 1=Low-5=High | 2.554 | 0.991 | 1 | 5 |
| Social status | 1=Low-5=High | 2.874 | 1.001 | 1 | 5 |
| Life satisfaction | 1=Unsatisfied-5=Satisfied | 3.635 | 1.056 | 1 | 5 |
| **Control variables** |  |  |  |  |  |
| Gender | 1=Male; 0=Female | 0.527 | 0.499 | 0 | 1 |
| Age | Year | 43.70 | 10.79 | 18 | 64 |
| Education of self | Level | 2.656 | 1.079 | 1 | 5 |
| Illiterate/semi-literate | 1=Yes; 0=No | 0.177 | 0.381 | 0 | 1 |
| Primary school | 1=Yes; 0=No | 0.230 | 0.421 | 0 | 1 |
| Junior high school | 1=Yes; 0=No | 0.406 | 0.491 | 0 | 1 |
| Senior high school | 1=Yes; 0=No | 0.134 | 0.341 | 0 | 1 |
| 3-Year college and above | 1=Yes; 0=No | 0.053 | 0.224 | 0 | 1 |
| Work for self/family | 1=Yes; 0=No | 0.565 | 0.496 | 0 | 1 |
| Medical insurance | Number of insurances | 0.956 | 0.318 | 0 | 5 |
| Pension insurance | Number of insurances | 0.651 | 0.587 | 0 | 5 |
| Family size | Number of family members | 4.489 | 1.825 | 2 | 17 |
| Education of spouse | Level | 2.642 | 1.108 | 1 | 5 |
| Illiterate/semi-literate | 1=Yes; 0=No | 0.188 | 0.391 | 0 | 1 |
| Primary school | 1=Yes; 0=No | 0.233 | 0.423 | 0 | 1 |
| Junior high school | 1=Yes; 0=No | 0.387 | 0.487 | 0 | 1 |
| Senior high school | 1=Yes; 0=No | 0.132 | 0.339 | 0 | 1 |
| 3-Year college and above | 1=Yes; 0=No | 0.060 | 0.237 | 0 | 1 |
| Education of father: Junior high school and above | 1=Yes; 0=No | 0.206 | 0.404 | 0 | 1 |
| Education of mother: Junior high school and above | 1=Yes; 0=No | 0.087 | 0.282 | 0 | 1 |
| **Regional fixed effect** |  |  |  |  |  |
| Eastern China | 1=Yes; 0=No | 0.484 | 0.500 | 0 | 1 |
| Central China | 1=Yes; 0=No | 0.278 | 0.448 | 0 | 1 |
| Western China | 1=Yes; 0=No | 0.238 | 0.426 | 0 | 1 |
| **Year fixed effect** |  |  |  |  |  |
| Year in 2012 | 1=Yes; 0=No | 0.317 | 0.465 | 0 | 1 |
| Year in 2016 | 1=Yes; 0=No | 0.339 | 0.474 | 0 | 1 |
| Year in 2018 | 1=Yes; 0=No | 0.343 | 0.475 | 0 | 1 |
| **Observations** | 10,647 |  |  |  |  |

Source: China Family Panel Studies (2012, 2016 and 2018). Note: ^a^ The depressive symptoms group is categorized using CES-D scores (depressive symptoms= CES-D of 16 or higher); ^b^ The instrumental variables are the community-level of family urban integration, where a community refers to the village in which the respondent lives.

Table S2 Family urban integration based on unsupervised machine learning clustering algorithm

| Variables | Years | 2012 | | 2016 | | 2018 | |
| --- | --- | --- | --- | --- | --- | --- | --- |
|  | Definition | Mean (%) | S.D. | Mean (%) | S.D. | Mean (%) | S.D. |
| Family urban integration | 1=Low; 2=Medium; 3=High | 1.130 | 0.393 | 1.372 | 0.595 | 1.400 | 0.583 |
| Family economic integration | 1=Low; 2=Medium; 3=High | 1.101 | 0.344 | 1.324 | 0.544 | 1.392 | 0.582 |
| Family income ^a^ | CNY | 54243 | 49693 | 81950 | 83967 | 98163 | 82231 |
| Family expense ^a^ | CNY | 49074 | 47509 | 83414 | 80521 | 83489 | 70257 |
| House expense/Family expense | % | 0.017 | 0.067 | 0.049 | 0.130 | 0.052 | 0.119 |
| Family social integration | 1=Low; 2=Medium; 3=High | 1.631 | 0.734 | 1.830 | 0.748 | 1.808 | 0.727 |
| Property owned by family members | 1=Yes; 0=No | 84.1% | 0.366 | 85.5% | 0.352 | 84.7% | 0.360 |
| Type of residential house | 1=Building; 0=Other | 61.0% | 0.488 | 56.8% | 0.495 | 47.0% | 0.499 |
| Collectively-distributed land | 1=None; 0=Other land | 25.1% | 0.434 | 21.1% | 0.408 | 22.6% | 0.419 |
| You are popular | 0=Lowest-10=Highest | 6.416 | 2.165 | 7.113 | 1.819 | 7.067 | 1.854 |
| Communist Party of China | 1=Yes; 0=No | 4.2% | 0.200 | 5.0% | 0.218 | 4.7% | 0.068 |
| Labor union | 1=Yes; 0=No | 0.8% | 0.089 | 4.5% | 0.206 | 5.0% | 0.219 |
| Association of individual workers | 1=Yes; 0=No | 0.4% | 0.060 | 4.1% | 0.198 | 5.5% | 0.229 |
| Performance of the government | 1=Worse-5=Good | 3.421 | 0.894 | 3.380 | 0.858 | 3.297 | 0.972 |
| Donation | 1=Yes; 0=No | 20.9% | 0.407 | 18.0% | 0.384 | 27.1% | 0.444 |
| Family psychological integration | 1=Low; 2=Medium; 3=High | 1.782 | 0.775 | 1.831 | 0.768 | 1.886 | 0.771 |
| Migrant spouse | 1=Yes; 0=No | 93.3% | 0.250 | 93.3% | 0.250 | 92.1% | 0.269 |
| Migrant children | 1=Yes; 0=No | 76.2% | 0.426 | 77.1% | 0.420 | 75.4% | 0.431 |
| Number of migrant children | Number | 1.105 | 0.832 | 1.152 | 0.855 | 1.133 | 0.862 |
| Have meals together as a family | Number | 3.888 | 1.625 | 3.911 | 1.729 | 3.949 | 1.749 |
| Trust neighbors | 0=Untrust-10=Trust | 6.416 | 2.165 | 6.612 | 2.186 | 6.725 | 2.020 |
| Feel lonely | 1=Most-4=Never | 3.729 | 0.588 | 3.668 | 0.632 | 3.606 | 0.658 |
| Observations |  | 3,377 | | 3,614 | | 3,656 | |

Source: China Family Panel Studies (2012, 2016 and 2018). Note: ^a^ Winsorization approach was adopted to solve outliers of family income and family expense.

Table S3 Effect of family urban integration on depressive symptoms of migrant workers

| Variables | Definition | Depressive symptoms (1=Yes; 0=No) | | | |
| --- | --- | --- | --- | --- | --- |
|  |  | Pooled Logit | | Pooled Logit with Controls | |
|  |  | (1) | (2) | (3) | (4) |
| Family economic integration | 1=Low; 2=Medium; 3=High | -0.173*** | -0.122*** | -0.107** | -0.074** |
|  |  | (0.046) | (0.047) | (0.048) | (0.031) |
| Family social integration | 1=Low; 2=Medium; 3=High | -0.267*** | -0.199*** | -0.276*** | -0.212*** |
|  |  | (0.033) | (0.034) | (0.034) | (0.008) |
| Family psychological integration | 1=Low; 2=Medium; 3=High | -0.294*** | -0.236*** | -0.290*** | -0.224*** |
|  |  | (0.032) | (0.033) | (0.035) | (0.036) |
| Family urban integration | 1=Low; 2=Medium; 3=High | -0.138*** |  | -0.085* |  |
|  |  | (0.043) |  | (0.044) |  |
| Control variables ^e^ |  | No | No | Yes | Yes |
| Regional fixed effect |  | No | No | Yes | Yes |
| Observations |  | 10,647 | 10,647 | 10,647 | 10,647 |

Source: China Family Panel Studies (2012, 2016 and 2018). Note: ^a^ ***/**/* Statistically significant at the 1%/5%/10% level; ^b^ Robust standard errors are in parentheses; ^c^ We estimate regressions weighting observations to give more weight to those less likely to remain in the sample (and thus attempt to restore the representativeness of the sample – Bai et al., 2022). Specifically, we calculate the wave number of participation in the survey, and then weight the regressions using the inverse of the calculated number; ^d^ Column (1) and column (3) contain four regression equations, which respectively show the impact of family urban integration and its three dimensions on depressive symptoms; Column (2) and column (4) contain one regression equation, which shows the overall impact of three dimensions of family urban integration on depressive symptoms. ^e^ Control variables include gender, age, age square, education, work for self/family, medical insurance, pension insurance, family size, and education of spouse, father and mother.

Table S4 Correlations between baseline variables and family urban integration (Order Probit)

| Variables  (Definition) | Family  economic integration | Family  social integration | Family  psychological integration | Family  urban integration |
| --- | --- | --- | --- | --- |
|  | (1=Low; 2=Medium; 3=High) | | | |
|  | (1) | (2) | (3) | (4) |
| Depressive symptoms | -0.142*** | -0.256*** | -0.250*** | -0.107*** |
| (1=Yes; 0=No) | (0.033) | (0.028) | (0.029) | (0.032) |
| **Panel A: Migrant Characteristics** |  |  |  |  |
| Gender | 0.044 | -0.041 | 0.090*** | 0.050* |
| (1=Male; 0=Female) | (0.032) | (0.025) | (0.026) | (0.030) |
| Age | 0.032*** | 0.008 | -0.044*** | 0.035*** |
| (Year) | (0.012) | (0.009) | (0.010) | (0.011) |
| Age2 | -0.000** | 0.000 | 0.001*** | -0.000*** |
| (Square) | (0.000) | (0.000) | (0.000) | (0.000) |
| Education of self | 0.166*** | 0.004 | 0.038*** | 0.137*** |
| (Level) | (0.017) | (0.014) | (0.014) | (0.017) |
| Work for self/family | 0.071** | 0.060** | 0.044* | 0.151*** |
| (1=Yes; 0=No) | (0.033) | (0.026) | (0.026) | (0.031) |
| Medical insurance | 0.057 | 0.053 | 0.123*** | 0.024 |
| (Number of medical insurees) | (0.052) | (0.040) | (0.042) | (0.051) |
| Pension insurance | 0.096*** | 0.070*** | 0.051** | 0.056** |
| (Number of pension insurees) | (0.028) | (0.022) | (0.022) | (0.027) |
| **Panel B: Household Characteristics** |  |  |  |  |
| Family size | 0.089*** | 0.000 | 0.289*** | 0.063*** |
| (Number of family members) | (0.008) | (0.007) | (0.009) | (0.007) |
| Education of spouse | 0.158*** | 0.009 | -0.005 | 0.138*** |
| (Level) | (0.017) | (0.014) | (0.014) | (0.016) |
| Education of father: Junior high | -0.005 | 0.049 | 0.005 | -0.001 |
| school and above (1=Yes; 0=No) | (0.040) | (0.033) | (0.033) | (0.038) |
| Education of mother: Junior high | 0.095* | -0.001 | 0.022 | 0.038 |
| school and above (1=Yes; 0=No) | (0.055) | (0.044) | (0.049) | (0.053) |
| Regional fixed effect | Yes | Yes | Yes | Yes |
| Year fixed effect | Yes | Yes | Yes | Yes |
| Migrant fixed effect | Yes | Yes | Yes | Yes |
| Observations | 10,647 | 10,647 | 10,647 | 10,647 |

Source: China Family Panel Studies (2012, 2016 and 2018). Note: ^a^ ***/**/* Statistically significant at the 1%/5%/10% level; ^b^ Robust standard errors are in parentheses; ^c^ We estimate regressions weighting observations to give more weight to those less likely to remain in the sample (and thus attempting to maintain the representativeness of the sample – Bai et al., 2022). Specifically, we calculate the wave number of migrant workers’ participation in the survey, and then weight the regressions using the inverse of the calculated number.

Table S5 Robustness Check: Effect of family urban integration on CES-D scores (Two-stage Least Squares)

| Variable | Definition | CES-D (scores) | | | |
| --- | --- | --- | --- | --- | --- |
|  |  | (1) | (2) | (3) | (4) |
| Family economic integration | 1=Low; 2=Medium; 3=High | -1.182*** |  |  |  |
|  |  | (0.450) |  |  |  |
| Family social integration | 1=Low; 2=Medium; 3=High |  | -2.763*** |  |  |
|  |  |  | (1.018) |  |  |
| Family psychological integration | 1=Low; 2=Medium; 3=High |  |  | -4.068** |  |
|  |  |  |  | (1.832) |  |
| Family urban integration | 1=Low; 2=Medium; 3=High |  |  |  | -1.039** |
|  |  |  |  |  | (0.479) |
| F-statistics |  | 139.05 | 21.97 | 131.17 | 105.31 |
| p-value |  | 0.000 | 0.000 | 0.000 | 0.000 |
| Control variables ^d^ |  | Yes | Yes | Yes | Yes |
| Regional fixed effect |  | Yes | Yes | Yes | Yes |
| Year fixed effect |  | Yes | Yes | Yes | Yes |
| Migrant fixed effect |  | Yes | Yes | Yes | Yes |
| Observations |  | 10,647 | 10,647 | 10,647 | 10,647 |

Source: China Family Panel Studies (2012, 2016 and 2018). Note: ^a^ ***/**/* Statistically significant at the 1%/5%/10% level; ^b^ Robust standard errors are in parentheses; ^c^ We estimate regressions weighting observations to give more weight to those less likely to remain in the sample (and thus attempting to maintain the representativeness of the sample – Bai et al., 2022). Specifically, we calculate the wave number of participation in the survey, and then weight the regressions using the inverse of the calculated number; ^d^ Control variables include gender, age, age square, education, work for self/family, medical insurance, pension insurance, family size, and education of spouse, father and mother.

Table S6 Robustness Check: Effect of family urban integration on CES-D scores (Quantile Regression)

| Variable  (Definition) | CES-D (scores) | | | | |
| --- | --- | --- | --- | --- | --- |
|  | 10% | 25% | 50% | 75% | 90% |
|  | (1) | (2) | (3) | (4) | (5) |
| Family economic integration | -0.250** | -0.250*** | -0.266*** | -0.560*** | -0.684** |
| (1=Low; 2=Medium; 3=High) | (0.127) | (0.074) | (0.100) | (0.200) | (0.285) |
| Family social integration | -1.000*** | -0.817*** | -1.256*** | -1.405*** | -1.508*** |
| (1=Low; 2=Medium; 3=High) | (0.105) | (0.079) | (0.115) | (0.145) | (0.204) |
| Family psychological integration | -0.622*** | -0.786*** | -1.176*** | -1.546*** | -2.015*** |
| (1=Low; 2=Medium; 3=High) | (0.117) | (0.089) | (0.124) | (0.148) | (0.210) |
| Family urban integration | -0.262* | -0.209* | -0.385*** | -0.566*** | -0.482* |
| (1=Low; 2=Medium; 3=High) | (0.142) | (0.108) | (0.127) | (0.192) | (0.248) |
| Control variables ^d^ | Yes | Yes | Yes | Yes | Yes |
| Regional fixed effect | Yes | Yes | Yes | Yes | Yes |
| Year fixed effect | Yes | Yes | Yes | Yes | Yes |
| Migrant fixed effect | Yes | Yes | Yes | Yes | Yes |
| Observations | 10,647 | 10,647 | 10,647 | 10,647 | 10,647 |

Source: China Family Panel Studies (2012, 2016 and 2018). Note: ^a^ ***/**/* Statistically significant at the 1%/5%/10% level; ^b^ Robust standard errors are in parentheses; ^c^ Column (1) contains four regression equations, which respectively show the impact of family urban integration and its three dimensions on CES-D scores. The same goes for the other columns; ^d^ Control variables include gender, age, age square, education, work for self/family, medical insurance, pension insurance, family size, and education of spouse, father and mother.

Table S7 Robustness Check: Effect of family urban integration on depressive symptoms (IVlasso)

| Variable | Definition | Depressive symptoms (1=Yes; 0=No) | | | |
| --- | --- | --- | --- | --- | --- |
|  |  | (1) | (2) | (3) | (4) |
| Family economic integration | 1=Low; 2=Medium; 3=High | -0.110*** |  |  |  |
|  |  | (0.030) |  |  |  |
| Family social integration | 1=Low; 2=Medium; 3=High |  | -0.217*** |  |  |
|  |  |  | (0.063) |  |  |
| Family psychological integration | 1=Low; 2=Medium; 3=High |  |  | -0.378*** |  |
|  |  |  |  | (0.099) |  |
| Family urban integration | 1=Low; 2=Medium; 3=High |  |  |  | -0.095*** |
|  |  |  |  |  | (0.032) |
| Control variables ^e^ |  | Yes | Yes | Yes | Yes |
| Regional fixed effect |  | Yes | Yes | Yes | Yes |
| Year fixed effect |  | Yes | Yes | Yes | Yes |
| Migrant fixed effect |  | Yes | Yes | Yes | Yes |
| Observations |  | 10,647 | 10,647 | 10,647 | 10,647 |

Source: China Family Panel Studies (2012, 2016 and 2018). Note: ^a^ ***/**/* Statistically significant at the 1%/5%/10% level; ^b^ Robust standard errors are in parentheses; ^c^ We estimate regressions weighting observations to give more weight to those less likely to remain in the sample (and thus attempting to maintain the representativeness of the sample – Bai et al., 2022). Specifically, we calculate the wave number of participation in the survey, and then weight the regressions using the inverse of the calculated number; ^d^ The instrumental variables are the community-level of family urban integration and its sub-indicators (see Table S1 for detailed indicator characteristics), and then use IVlasso to select appropriate instrumental variables for regression; ^e^ Control variables include gender, age, age square, education, work for self/family, medical insurance, pension insurance, family size, and education of spouse, father and mother.

Table S8 Intermediate outcomes and depressive symptoms (KHB method)

| Variable | Explanatory variables (1=Low; 2=Medium; 3=High) | | |
| --- | --- | --- | --- |
|  | Family economic integration | Family social integration | Family psychological integration |
|  | (1) | (2) | (3) |
| Total effects | -0.187*** | -0.308*** | -0.320*** |
|  | (0.050) | (0.034) | (0.036) |
| Direct effects | -0.156*** | -0.265*** | -0.251*** |
|  | (0.050) | (0.035) | (0.036) |
| Indirect effects | -0.030*** | -0.043*** | -0.069*** |
|  | (0.006) | (0.006) | (0.009) |
| Control variables ^c^ | Yes | Yes | Yes |
| Regional fixed effect | Yes | Yes | Yes |
| Year fixed effect | Yes | Yes | Yes |
| Migrant fixed effect | Yes | Yes | Yes |
| Observations | 10,647 | 10,647 | 10,647 |

Source: China Family Panel Studies (2012, 2016 and 2018). Note: ^a^ ***/**/* Statistically significant at the 1%/5%/10% level; ^b^ Robust standard errors are in parentheses; ^c^ Control variables include gender, age, age square, education, work for self/family, medical insurance, pension insurance, family size, and education of spouse, father and mother.

Table S9 Heterogeneity Analysis: Effect of family urban integration on depressive symptoms based on gender (Pooled IVprobit)

| Variable | Definition | Depressive symptoms (1=Yes; 0=No) | | Diff |
| --- | --- | --- | --- | --- |
|  |  | (1) | (2) | (2)-(1) |
| Family economic integration | 1=Low; 2=Medium; 3=High | -0.206* | -0.373*** | -0.167*** |
|  |  | (0.119) | (0.113) |  |
| Family social integration | 1=Low; 2=Medium; 3=High | -0.397* | -0.586*** | -0.189*** |
|  |  | (0.230) | (0.206) |  |
| Family psychological integration | 1=Low; 2=Medium; 3=High | -0.156 | -0.944** | -0.788*** |
|  |  | (0.376) | (0.389) |  |
| Family urban integration | 1=Low; 2=Medium; 3=High | -0.189 | -0.228* | -0.039*** |
|  |  | (0.120) | (0.122) |  |
| Gender |  | Male | Female |  |
| Control variables ^d^ |  | Yes | Yes |  |
| Regional fixed effect |  | Yes | Yes |  |
| Year fixed effect |  | Yes | Yes |  |
| Migrant fixed effect |  | Yes | Yes |  |
| Observations |  | 5,612 | 5,035 |  |

Source: China Family Panel Studies (2012, 2016 and 2018). Note: ^a^ ***/**/* Statistically significant at the 1%/5%/10% level; ^b^ Robust standard errors are in parentheses; ^c^ Column (1) contain four regression equations, which respectively show the impact of family urban integration and its three dimensions on depressive symptoms. The same goes for column (2); ^d^ Control variables include gender, age, age square, education, work for self/family, medical insurance, pension insurance, family size, and education of spouse, father and mother.

Table S10 Heterogeneity Analysis: Effect of family urban integration on depressive symptoms based on age (Pooled IVprobit)

| Variable | Definition | Depressive symptoms (1=Yes; 0=No) | | Diff |
| --- | --- | --- | --- | --- |
|  |  | (1) | (2) | (2)-(1) |
| Family economic integration | 1=Low; 2=Medium; 3=High | -0.439*** | -0.100 | 0.339*** |
|  |  | (0.114) | (0.117) |  |
| Family social integration | 1=Low; 2=Medium; 3=High | -0.468** | -0.533* | -0.065*** |
|  |  | (0.183) | (0.283) |  |
| Family psychological integration | 1=Low; 2=Medium; 3=High | -0.743** | -0.185 | 0.558*** |
|  |  | (0.350) | (0.406) |  |
| Family urban integration | 1=Low; 2=Medium; 3=High | -0.378*** | 0.036 | 0.414*** |
|  |  | (0.116) | (0.127) |  |
| Age ^d^ |  | First-generation | New-generation |  |
| Control variables ^e^ |  | Yes | Yes |  |
| Regional fixed effect |  | Yes | Yes |  |
| Year fixed effect |  | Yes | Yes |  |
| Migrant fixed effect |  | Yes | Yes |  |
| Observations |  | 7,087 | 3,560 |  |

Source: China Family Panel Studies (2012, 2016 and 2018). Note: ^a^ ***/**/* Statistically significant at the 1%/5%/10% level; ^b^ Robust standard errors are in parentheses; ^c^ Column (1) contain four regression equations, which respectively show the impact of family urban integration and its three dimensions on depressive symptoms. The same goes for column (2); ^d^ Researchers have distinguished two generations of migrant workers, that is, first-generation migrant workers who were born before 1980 and new-generation migrant workers who were born in or after 1980 (Zhang et al., 2022); ^e^ Control variables include gender, age, age square, education, work for self/family, medical insurance, pension insurance, family size, and education of spouse, father and mother.

Table S11 Heterogeneity Analysis: Effect of family urban integration on depressive symptoms based on education (Pooled IVprobit)

| Variable  (Definition) | Depressive symptoms (1=Yes; 0=No) | | | Diff | Diff |
| --- | --- | --- | --- | --- | --- |
|  | (1) | (2) | (3) | (2)-(1) | (3)-(1) |
| Family economic integration | -0.655*** | -0.275** | -0.029 | 0.380*** | 0.626*** |
| (1=Low; 2=Medium; 3=High) | (0.167) | (0.128) | (0.151) |  |  |
| Family social integration | -0.443* | -0.413* | -0.807* | 0.030*** | -0.364*** |
| (1=Low; 2=Medium; 3=High) | (0.232) | (0.235) | (0.426) |  |  |
| Family psychological integration | -0.971*** | -0.027 | -0.559 | 0.944*** | 0.412*** |
| (1=Low; 2=Medium; 3=High) | (0.367) | (0.394) | (1.048) |  |  |
| Family urban integration | -0.589*** | -0.183 | 0.146 | 0.406*** | 0.735*** |
| (1=Low; 2=Medium; 3=High) | (0.161) | (0.134) | (0.170) |  |  |
| Education | Primary and below | Junior high school | Senior and above |  |  |
| Control variables ^d^ | Yes | Yes | Yes |  |  |
| Regional fixed effect | Yes | Yes | Yes |  |  |
| Year fixed effect | Yes | Yes | Yes |  |  |
| Migrant fixed effect | Yes | Yes | Yes |  |  |
| Observations | 4,334 | 4,322 | 1,991 |  |  |

Source: China Family Panel Studies (2012, 2016 and 2018). Note: ^a^ ***/**/* Statistically significant at the 1%/5%/10% level; ^b^ Robust standard errors are in parentheses; ^c^ Column (1) contains four regression equations, which respectively show the impact of family urban integration and its three dimensions on depressive symptoms. The same goes for column (2) and column (3); ^d^ Control variables include gender, age, age square, education, work for self/family, medical insurance, pension insurance, family size, and education of spouse, father and mother.

Table S12 CES-D (20-item) in 2012, 2016 and 2018

| Item | During the past week: | Score ^a^ | | | |
| --- | --- | --- | --- | --- | --- |
|  |  | A | B | C | D |
|  |  |  |  |  |  |
| 1 | I was bothered by things that usually don’t bother me. | 0 | 1 | 2 | 3 |
| 2 | I did not feel like eating; my appetite was poor. | 0 | 1 | 2 | 3 |
| 3 | I felt that I couldn’t shake off the blues even with help from my family or friends. | 0 | 1 | 2 | 3 |
| 4 | I felt I was just as good as other people. | 3 | 2 | 1 | 0 |
| 5 | I had trouble keeping my mind on what I was doing. | 0 | 1 | 2 | 3 |
| 6 | I felt depressed. | 0 | 1 | 2 | 3 |
| 7 | I felt that everything I did was an effort. | 0 | 1 | 2 | 3 |
| 8 | I felt hopeful about the future. | 3 | 2 | 1 | 0 |
| 9 | I thought my life had been a failure. | 0 | 1 | 2 | 3 |
| 10 | I felt fearful. | 0 | 1 | 2 | 3 |
| 11 | My sleep was restless. | 0 | 1 | 2 | 3 |
| 12 | I was happy. | 3 | 2 | 1 | 0 |
| 13 | I talked less than usual. | 0 | 1 | 2 | 3 |
| 14 | I felt lonely. | 0 | 1 | 2 | 3 |
| 15 | People were unfriendly. | 0 | 1 | 2 | 3 |
| 16 | I enjoyed life. | 3 | 2 | 1 | 0 |
| 17 | I had crying spells. | 0 | 1 | 2 | 3 |
| 18 | I felt sad. | 0 | 1 | 2 | 3 |
| 19 | I felt that people disliked me. | 0 | 1 | 2 | 3 |
| 20 | I could not get going. | 0 | 1 | 2 | 3 |

Source: CFPS (2012, 2016 and 2018)

Note: ^a^ A means “Rarely or none of the time (less than 1 day)”; B means “Some or a little of the time (1-2 days)”; C means “Occasionally or a moderate amount of time (3-4 days)”; D means “Most or all of the time (5-7 days)”. ^b^ The score is the sum of the 20 questions. Among them, items 4, 8, 12 and 16 represent positive emotions, and the scores need to be reversed and then summed. Possible range is 0-60.


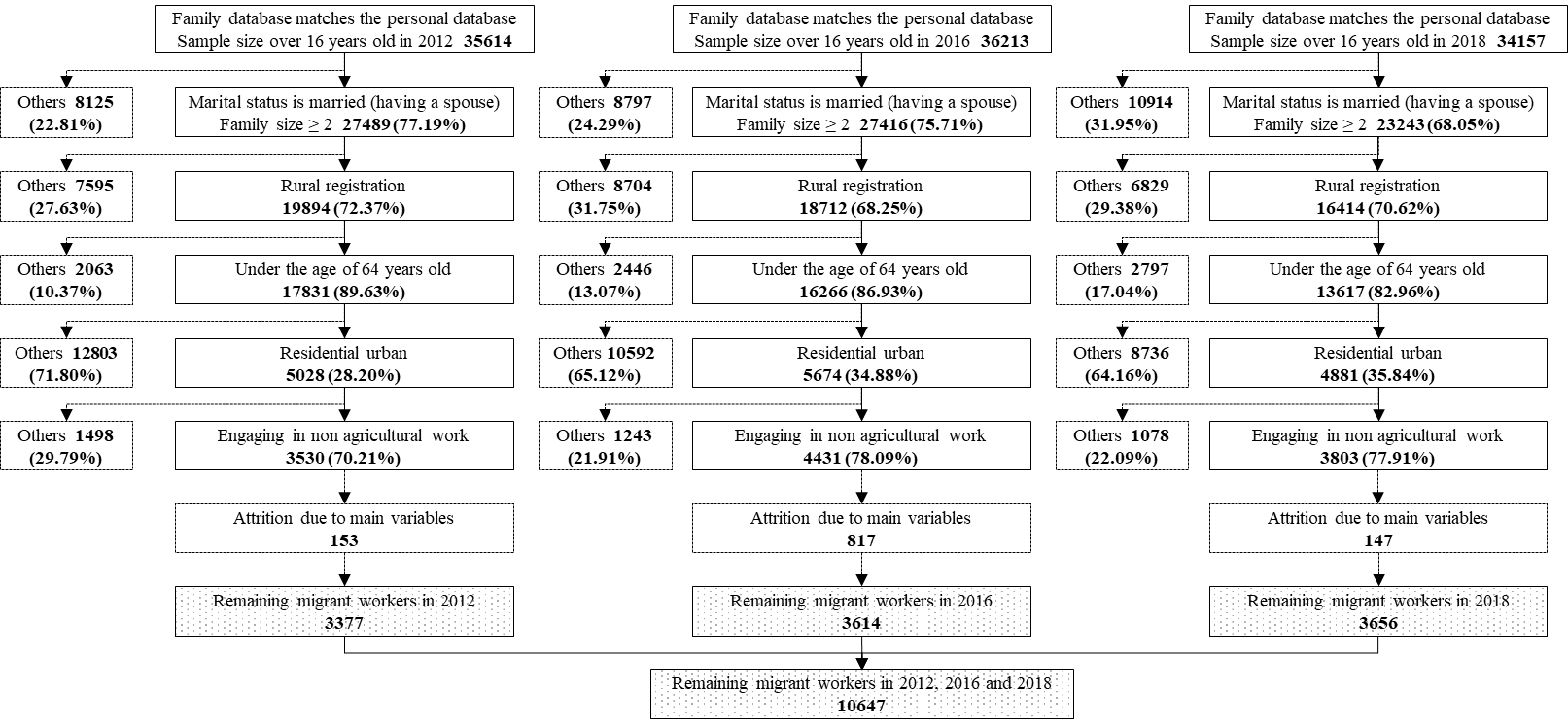


Note: Solid lines represent retained samples; Dashed lines represent unused samples; Shadow represents the sample used for final analysis.

Figure S1 Data selection detailed procedure


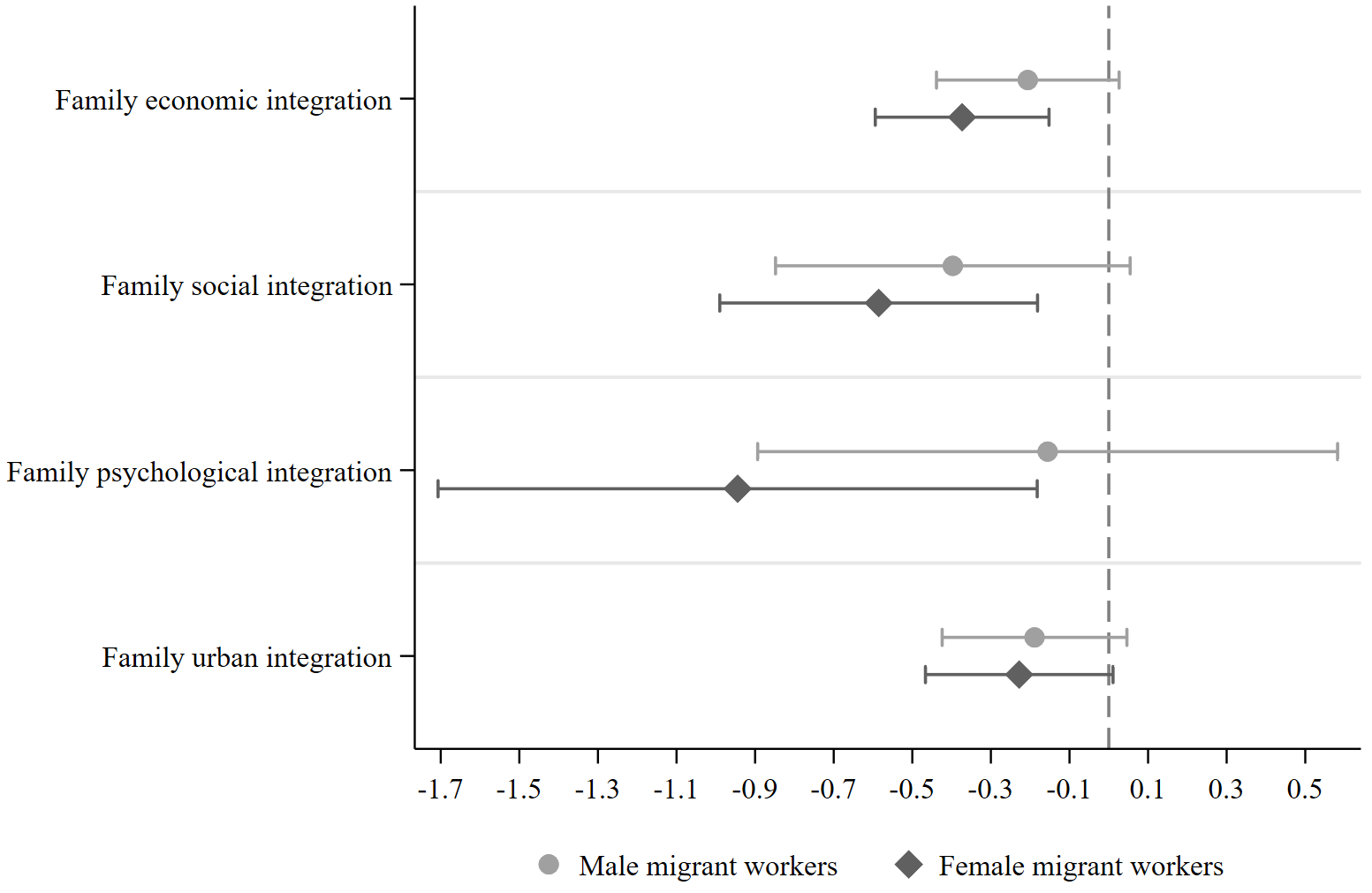


Figure S2 Heterogeneity analysis based on gender


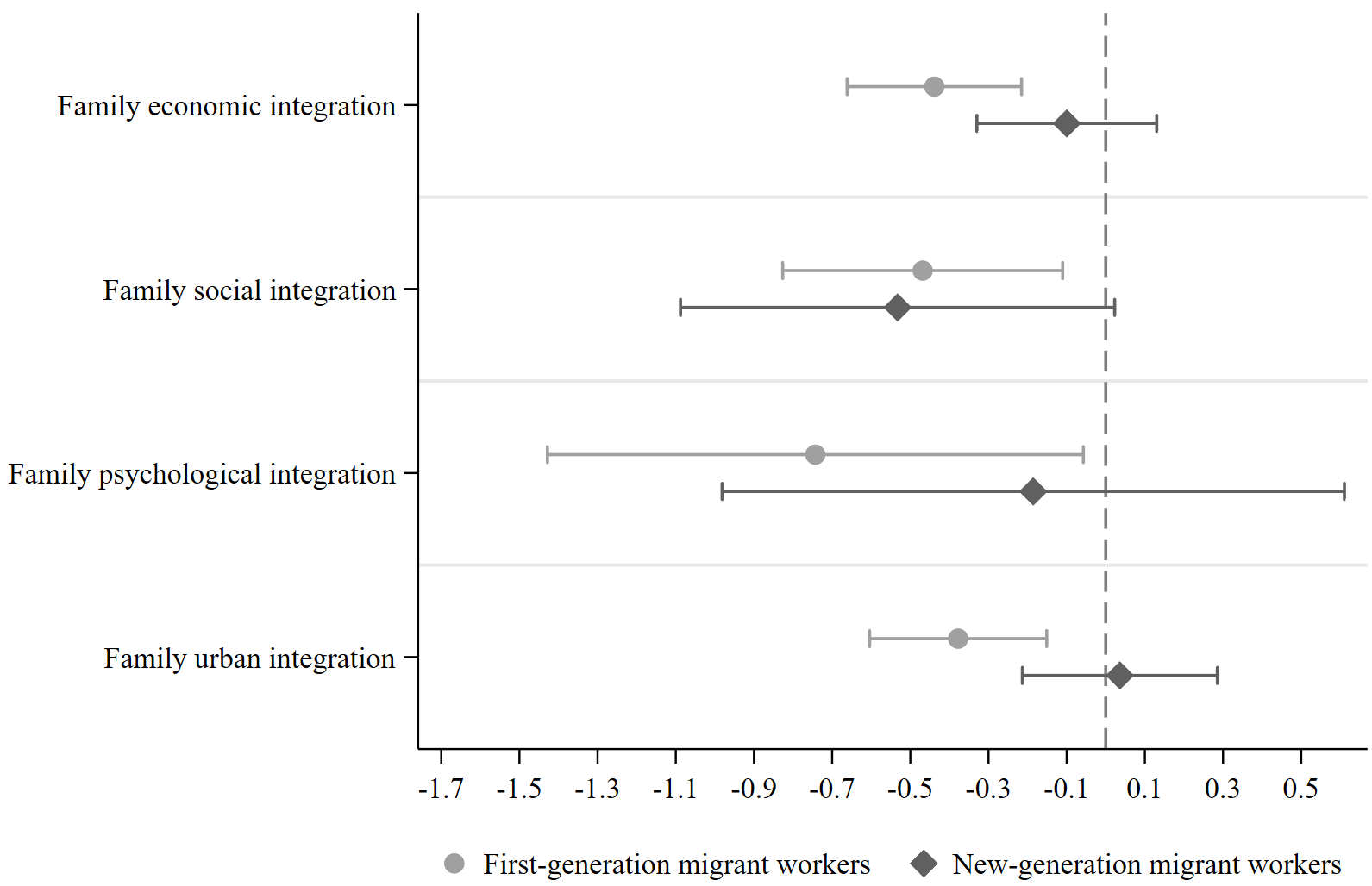


Figure S3 Heterogeneity analysis based on age


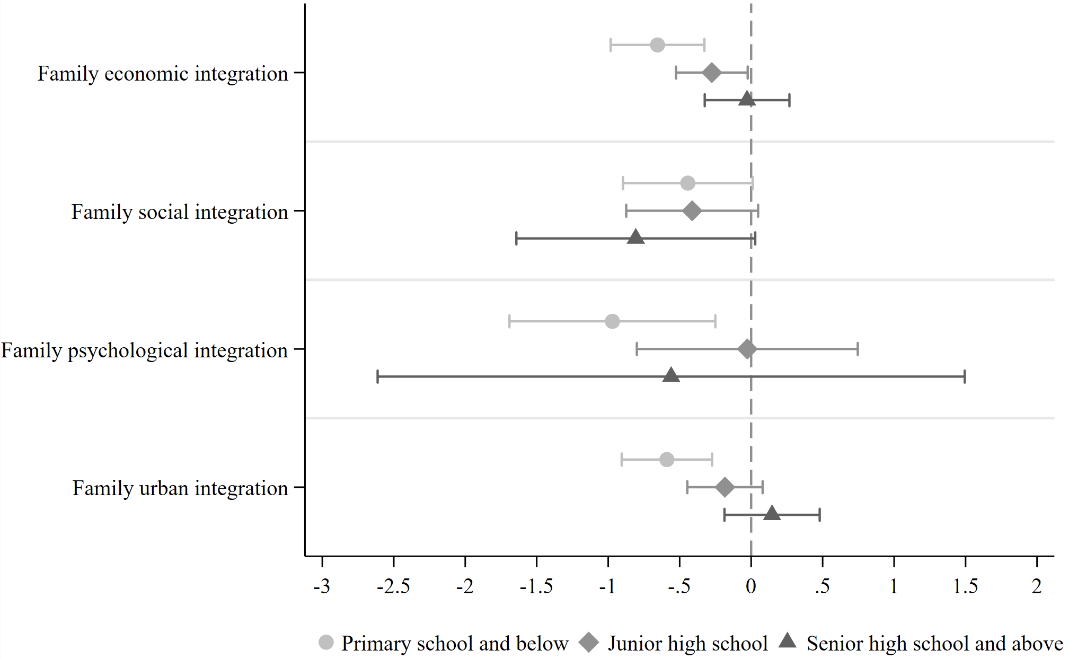


Figure S4 Heterogeneity analysis based on education
